# Energetic and Kinetic Origins of CALB Interfacial Activation Revealed by PaCS-MD/MSM

**DOI:** 10.1021/acs.jpcb.3c02041

**Published:** 2023-08-10

**Authors:** Tegar N. Wijaya, Akio Kitao

**Affiliations:** † School of Life Science and Technology, Tokyo Institute of Technology. 2-12-1 Ookayama, Meguro-ku, Tokyo 152-8550, Japan; ‡ Department of Chemistry, Universitas Pertamina, Jl. Teuku Nyak Arief, Simprug, Jakarta 12220, Indonesia

## Abstract

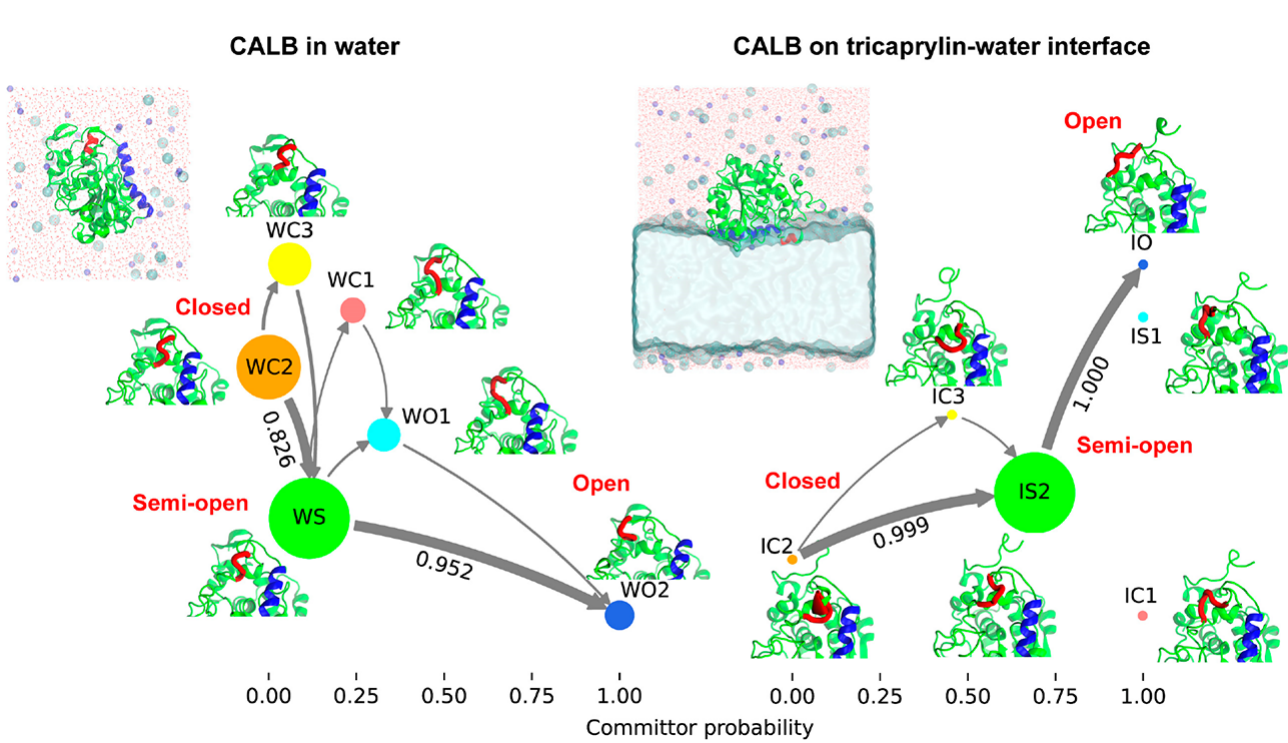

The conformational dynamics of *Candida antarctica* lipase B (CALB) was investigated by molecular dynamics (MD) simulation, parallel cascade selection MD (PaCS-MD), and the Markov state model (MSM) and mainly focused on the lid-opening motion closely related to substrate binding. All-atom MD simulation of CALB was conducted in water and on the interface of water and tricaprylin. CALB initially situated in water and separated by layers of water from the interface is spontaneously adsorbed onto the tricaprylin surface during MD simulation. The opening and closing motions of the lid are simulated by PaCS-MD, and subsequent MSM analysis provided the free-energy landscape and time scale of the conformational transitions among the closed, semiopen, and open states. The closed state is the most stable in the water system, but the stable conformation in the interface system shifts to the semiopen state. These effects could explain the energetics and kinetics origin of the previously reported interfacial activation of CALB. These findings could help expand the application of CALB toward a wide variety of substrates.

## Introduction

Lipase is a class of enzymes with wide applications in industry.^1^ Lipase catalyzes various reactions such as hydrolysis,^2,3^ esterification,^4,5^ and transesterification.^6,7^ Central to lipase activity is interfacial activation,^8^ which enhances activity upon adsorption on the interface between water and lipids. *Candida antarctica* lipase B (CALB) is one of the most widely used lipases in industry and academia^9^ and is utilized in various applications such as biodiesel production^10,11^ and chemical synthesis.^12,13^

**Figure 1:**
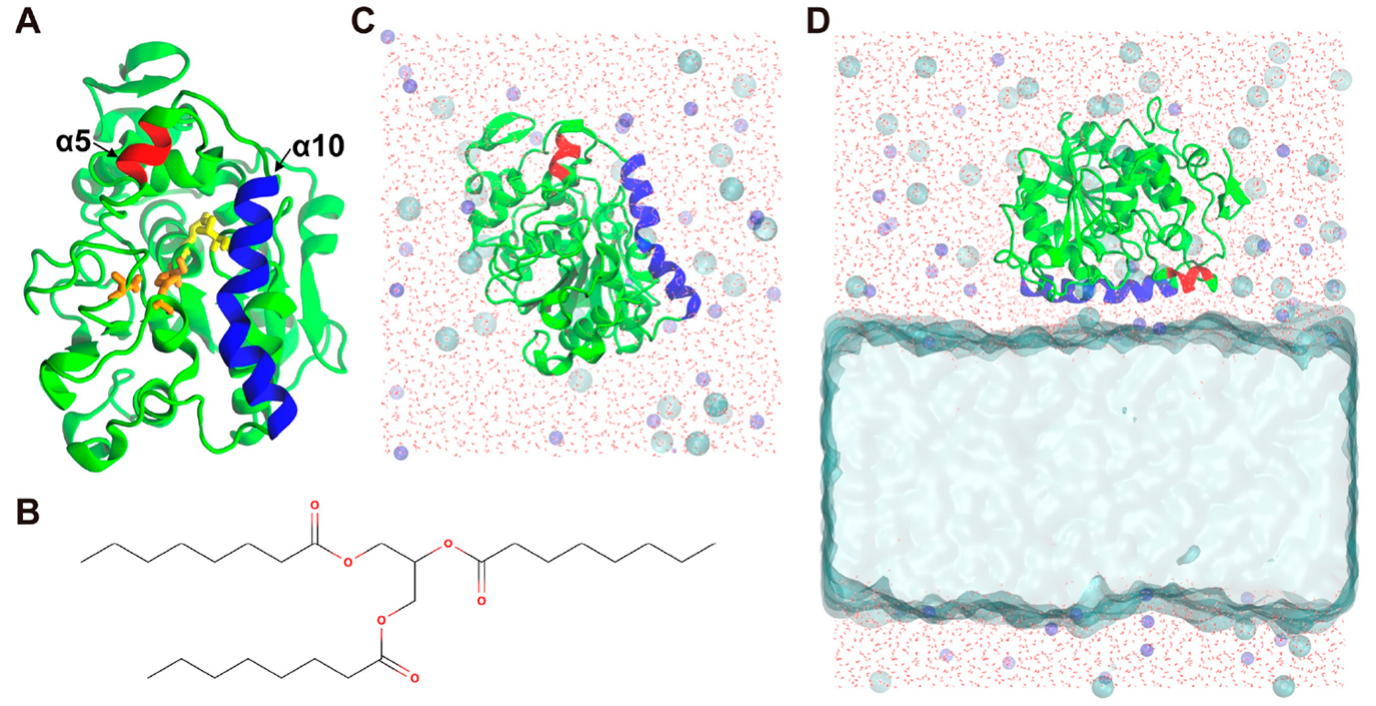
A. Three-dimensional structure of CALB (PDB id: 1TCA) with a new cartoon representation. The α5 lid region (residues 142–146), α10 lid region (residues 268–287), the catalytic triad (Ser105, Asp187, and His224), and the oxyanion hole (Thr40 and Gln106) are colored red, blue, orange, and yellow, respectively. B. Chemical structure of tricaprylin. C and D. Initial configurations in the molecular dynamics simulation for CALB in water (CALB/W) and on the tricaprylin–water interface (CALB/I), respectively. Blue and cyan spheres represent Na^+^ and Cl^–^ ions, respectively. Molecular images in this article were created using visual molecular dynamics (VMD).^26^

The three-dimensional structure of CALB has been resolved by X-ray crystallography.^14^ The structure shows that a catalytic triad comprising Ser105, Asp187, and His224 is located behind a lid region consisting of two α helices, the small α5 (residues 142–146) helix and the large α10 (residues 268–287) helix (Figure 1A).

The interfacial activation of lipases was observed for different types of hydrophobic surfaces, e.g., the substrate^15,16^ and solid surfaces.^17,18^ An early study showed that CALB does not exhibit significant interfacial activation compared to other lipases.^16^ However, a later report indicated the interfacial activation of CALB immobilized on a highly hydrophobic surface toward a bulky substrate.^18^ Interfacial activation is controlled by conformational dynamics of the lid region, especially by the α5 helix, as indicated by the open and closed conformations of the crystal structures of CALB.^19^ Additionally, several molecular dynamics studies showed that the α5 helix is a flexible region which could facilitate lid dynamics.^20−22^ In addition, the α5 helix also influences the substrate selectivity as shown by “lid-swapping” experiments with the homologues.^23^ Therefore, understanding the dynamics of the lid region and its interaction with the hydrophobic surface is important to understanding the mechanisms of substrate binding and catalysis.

Molecular dynamics (MD) simulation is a powerful molecular simulation method for studying complex molecular systems at the atomic level. Several MD studies have been conducted to investigate the interfacial activation of CALB. A conventional MD (CMD) simulation study performed to construct the free-energy landscape of CALB in water showed the existence of close, crystal-like, and open conformations with similar free energy values.^21^ In contrast, a lower probability for the open conformation in water was reported by replica exchange MD of CALB.^18^ Another study using CMD simulation indicated that interfacial activation is due to a lamellar-like nanostructure formed by triglycerides (tricaproin and tricaprylin) in a water–triglycerides system.^24^ Furthermore, the role of the conformational dynamics of CALB during substrate binding was investigated by MD and the Markov state model (MSM).^25^

Improvements should be achieved by employing enhanced conformational sampling methods with explicit solvent models for the hydrophobic surface, followed by analysis of the free-energy landscape concerning the lid open–close motion. In this regard, the combination of parallel cascade selection molecular dynamics (PaCS-MD),^27^ an enhanced sampling method, and the Markov state model (MSM),^28−30^ namely, PaCS-MD/MSM, can be used. In PaCS-MD, the efficient sampling of conformational changes is achieved by iteratively performing cycles of parallel short MD simulations. In each cycle, the initial structures are selected based on a collective variable describing the progression of conformational changes of interest, and MD runs are restarted with reinitialized velocities, effectively enhancing the probabilities of occurrence toward the expected conformational changes. Typically, the number of MD simulations conducted in parallel (the number of replicas, *n*_rep_) is 10–100, and the length of MD in each cycle is subnanoseconds to nanosceconds.^27,31^ In the next step, PaCS-MD trajectories are used as input to build an MSM. The free-energy landscape and some kinetic parameters of the conformational dynamics can be obtained from the MSM. The PaCS-MD/MSM combination has been used to study protein dynamics^32−34^ and protein–ligand,^34−36^ protein–protein,^37−39^ and protein–DNA interactions.^40^

In this article, we employ tricaprylin (Figure 1B) as a bulky model molecule to construct the hydrophobic surface. Conformational dynamics of CALB in water (hereafter CALB/W) and on the interface of water and tricaprylin (CALB/I) was investigated by MD and PaCS-MD/MSM, which allows direct observation of the effects of tricaprylin on the free-energy surface and kinetic properties of CALB conformational dynamics.

## Methods

### Parameterization of Tricaprylin

The atom types and force field parameters for tricaprylin were adopted from the AMBER lipid14 force field.^41^ All quantum calculations were performed using the Gaussian 16 software package,^42^ while topology generation and subsequent MD simulations were conducted using the AMBER package^43^ except where otherwise stated. The initial conformation of the tricaprylin monomer was constructed by the molefacture plugin of visual molecular dynamics (VMD) software,^26^ and geometry optimization and electrostatic potential (ESP) calculations were performed. To determine the atomic partial charges of the tricaprylin monomer, the RESP (restrained electrostatic potential) procedure^44^ at the B3LYP/6-31G* level of theory was employed. Atomic partial charges were fitted using *Antechamber*.^45^ Then, an ensemble of tricaprylin conformations was obtained from a 60 ns MD simulation of 144 tricaprylin molecules in the liquid phase at 298 K and 1 atm. For each of the 144 conformers of tricaprylin taken from the last snapshot of the MD trajectory, ESPs were calculated, and the partial charges were determined using the aforementioned procedure. The final atomic partial charges were obtained by averaging. To examine the obtained force field, a 50 ns MD simulation of a tricaprylin in the gas phase and that of 144 tricaprylins in the liquid phase were conducted. The obtained density and enthalpy of vaporization were 0.954 kg·L^–1^ and 144.4 kJ mol^–1^, which reproduced the experimental values of 0.954 kg·L^–1^^46^ and 135.4 kJ mol^–1^,^47^ respectively. This parameter set for tricaprylin was used in the following simulations.

### CMD Simulation of CALB in Water

To construct the CALB/W system, the crystal structure of CALB taken from the Protein Data Bank (PDB id: 1TCA) was protonated and solvated with 12 930 OPC water molecules^48^ into a cubic box using the tleap module of the Amber package.^43^ H++^49,50^ was employed to determine the protonation state of charged residues at pH 7. We also conducted p*K*_a_ prediction by PROPKA^51^ and found that the obtained result for His224 was not in agreement with the H++ result. We also searched possible roles of His224 in catalytic mechanisms and adopted a single protonated HIS at the Nε position, which was proposed as the His224 protonation of the substrate-free state in the catalytic cycle of lipases.^12^ We would also expect that the protonation of His224 does not significantly affect the results of this article because His224 was not identified as key residues for the open–close transition. However, the protonation of this histidine should be carefully examined if catalytic mechanisms are investigated. The system was neutralized, and additional NaCl ions were added to reproduce a 0.15 M NaCl solution. The Amber FF19SB force field^52^ was used to describe the protein. The constructed CALB/W system (Figure 1C) was energy minimized using the steepest-descent method to remove bad contacts between atoms introduced during the preparation of the model. The minimized system was equilibrated at 298.15 K using the Langevin thermostat with a collision frequency of 5 ps^–1^ for 500 ps, while the volume of the system was kept constant. During equilibration, positional restraints were applied to the Cα atoms of the protein with a force constant of 10 kcal mol^–1^ Å^–2^. The system was then brought to the correct density by isotropically performing a 500 ps NPT equilibration (wherein the number of particles, pressure, and temperature are all constant) run at 298.15 K and 1 atm using the Langevin thermostat with a collision frequency of 5 ps^–1^ and a Berendsen barostat with a relaxation time of 5 ps while keeping the positional restraints. The following preproduction run was performed without the positional restraints at 298.15 K and 1 atm using the Langevin thermostat with a collision frequency of 5 ps^–1^ and a Monte Carlo barostat for 500 ps. Finally, 10 independent production runs were performed for 500 ns using the same settings as for the preproduction run. After equilibration, the length of the box edge was approximately 7.5 nm.

The following procedure was common for both CALB/W and CALB/I. During the equilibration and production runs, the long-range electrostatic interactions were calculated by the particle mesh Ewald (PME) method, and the real-space nonbonded cutoff was made at 1 nm. All of the procedures above were conducted using the GPU-capable pmemd module of AMBER20.^43^

### MD Simulation of CALB in the Tricaprylin–Water Interface System

The tricaprylin–water interface was constructed by a method similar to those employed in the previous reports.^24,53^ First, 144 tricaprylin and 3806 water molecules were randomly inserted into a cubic box using Packmol^54^ to prepare the mixture of tricaprylin and water. The number of each molecule type was chosen so that the volumes of tricaprylin and water would be the same. The system was energy minimized and then equilibrated using the NVT ensemble with the V-rescale method at 300 K for 15 ns. Phase separation spontaneously occurred during this step. Then, the *z* direction was selected as the direction of phase separation, and the system was equilibrated for 50 ns using the NPAT ensemble at 300 K and 1 atm with the V-rescale method and the Berendsen barostat, keeping the box size along the *x* and *y* directions constant (4.5 × 5.2 nm^2^) while the box size along the *z* direction was free to change. In the next step, the equilibrated interface system was duplicated along the *x* and *y* directions (8.9 × 10.4 nm^2^). The large interface system was further equilibrated for 50 ns by using the NPAT ensemble at 298 K and 1 atm. To insert CALB into the water phase of the tricaprylin–water interface system, water molecules were removed, except for those situated within 0.3 nm of the tricaprylin molecules. Then CALB and the surrounding water molecules were taken from the equilibrated CALB/W system and placed 0.7 nm above the tricaprylin surface so that the lid faced the interface. Finally, the system was resolvated with water and 0.15 M NaCl. The above preparation method was conducted using the GROMACS 2019 package.^55^ The final CALB/I system contained 576 trycaprylin and 21 391 water molecules.

The CALB/I system (Figure 1D) was energy minimized and equilibrated for 1.0 ns under isothermal–isochoric conditions at 298.15 K using the Langevin thermostat with a collision frequency of 5 ps^–1^, imposing positional restraints onto the Cα atoms of the protein and the heavy atoms of tricaprylin with a force constant of 10 kcal mol^–1^ Å^–2^. The system was then equilibrated for 1.0 ns with the NPAT ensemble at 298.15 K and 1 atm using a Langevin thermostat with a collision frequency of 5 ps^–1^ and a Monte Carlo barostat while keeping the positional restraints. During this step, the box size along the *x* and *y* directions was kept constant while the box size along the *z* coordinate was free to move. A preproduction run was then performed with the NPAT ensemble without the positional restraints at 298.15 K and 1 atm using the Langevin thermostat with a collision frequency of 5 ps^–1^ and a Monte Carlo barostat for 1.0 ns. Corresponding to CALB/W, 10 independent production runs were performed for 500 ns using the same settings as for the preproduction run. After equilibration, the box size was around 8.9 × 10.4 × 12.6 nm^3^.

### PaCS-MD Procedure

The closed structure of both systems was chosen as the input for PaCS-MD. The initial closed structure of CALB/W was selected directly from the last snapshot of the 9th CMD run because during this run CALB stayed in the closed conformation for the longest time among the 10 production runs and stayed there until the end of the run. However, the initial closed structure of CALB/I could not be obtained directly because all 10 CMD did not run sampled closed conformations adequately in the case of CALB/I. Therefore, a single trial of PaCS-MD which consisted of 200 cycles was performed according to the cPaCS-MD procedure, as explained later. The input for this single trial was chosen from the last snapshot of the fifth CMD run, as this showed the smallest opening of the lid region among all CALB/I CMD runs. The snapshot from the last cycle was used as the initial closed structure for CALB/I. The obtained initial closed structure of both systems was used in the subsequent PaCS-MD procedure.

**Figure 2:**
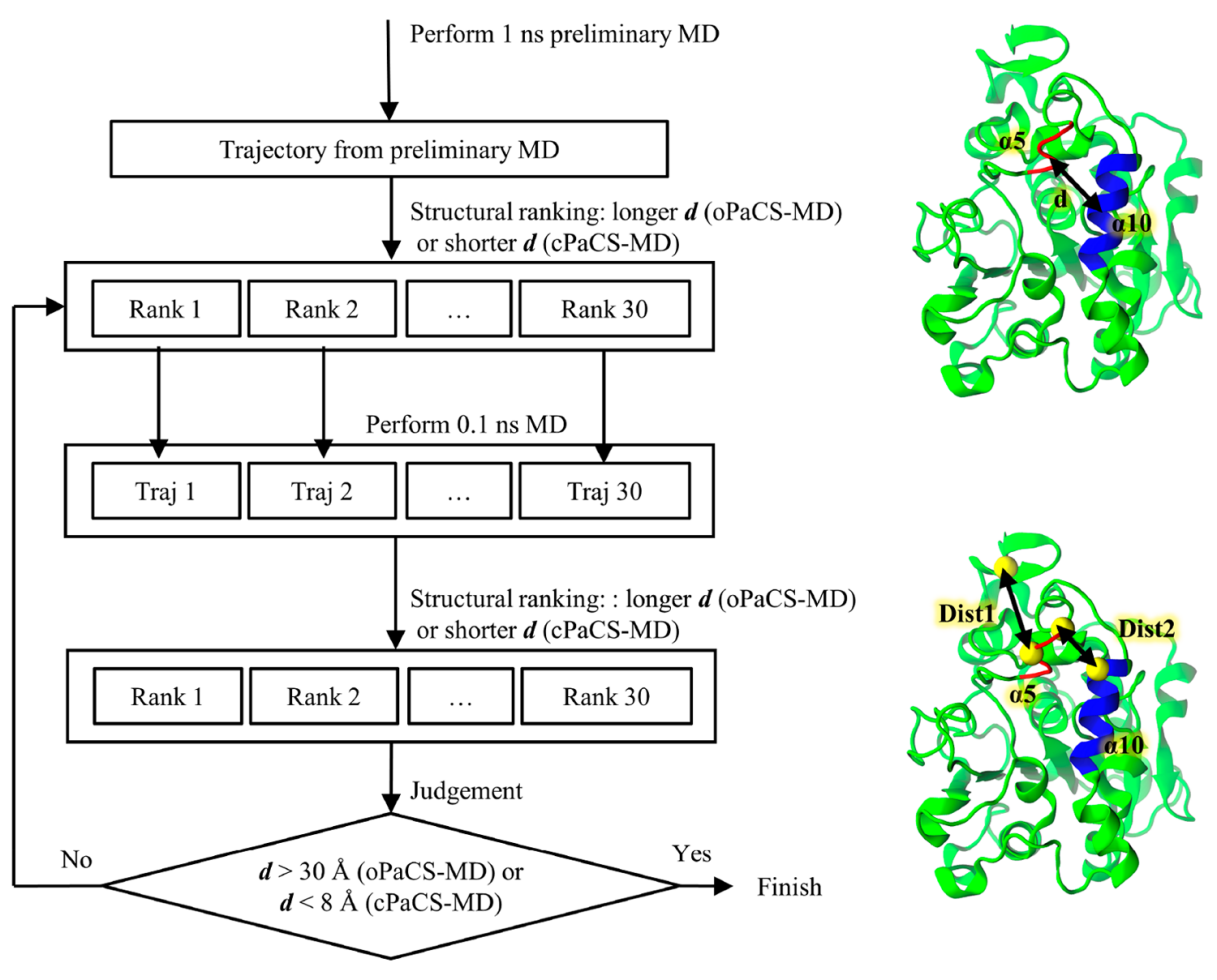
PaCS-MD procedures (left panel) and parameters (right top panel). MSM features (Dist1 and Dist2, right bottom panel) are also shown.

For both CALB/W and CALB/I, PaCS-MD simulations were performed in two stages. After preliminary MD to generate multiple initial structures, PaCS-MD was performed to sample conformational dynamics from the closed to open conformation, which here we call oPaCS-MD. In the second stage, open to closed conformational dynamics were sampled starting from the end snapshots from oPaCS-MD. The second stage is denoted as cPaCS-MD. A more detailed PaCS-MD procedure in each stage is pictured in Figure 2. The interlid distance ***d*** used in the structural ranking of the PaCS-MD trajectories is defined as the intercenter of mass distance between the α5 helix (residues 142–146) and half of the α10 helix (residues 278–287). The length of MDs in each PaCS-MD cycle was 0.1 ns, and *n*_rep_ was 30. First, a 1 ns preliminary MD simulation was performed, followed by the selection of the top 30 structures with longer ***d*** as the initial structures for the first cycle of oPaCS-MD. After 0.1 ns MD simulations, a structural ranking from the obtained trajectories and a selection of the initial structures for the next cycle were performed. This cycle was repeated until ***d*** became longer than 3.0 nm. Then, the simulation was switched to cPaCS-MD by selecting snapshots with shorter ***d*** until it reached 0.8 nm. One trial of oPaCS-MD or cPaCS-MD can sample only relatively limited conformational space along one transition pathway and neighborhood. To sufficiently sample different transition pathways between the closed and open states, this procedure was conducted multiple times until the conformational pathways between the closed and open states were sufficiently sampled. Since each MD simulation in PaCS-MD is conducted with a force field without any extra potential or force, each MD trajectory does not contain bias, but a set of raw PaCS-MD trajectories can contain statistical “bias” introduced by the selection. The possible bias in the PaCS-MD trajectories was corrected by MSM when the transition probability matrix (eq 1) was estimated. The combination of PaCS-MD and MSM was compared to other calculation methods and well examined with different conditions.^35,37^ In this article, we followed the standard procedures examined in the preceding works.

Table 1 shows the overview of the conducted CMDs and PaCS-MDs. The number of oPaCS-MD trials conducted for CALB/W and CALB/I was 20 and 5, respectively. After each trial of oPaCS-MD, two trials of cPaCS-MD were continued with different initial velocities. The simulation cost of PaCS-MD per trial is defined as 0.1 ns × the number of cycles × *n*_rep_. The total PaCS-MD simulation costs for CALB/W and CALB/I were 5.60 and 8.89 μs, respectively.

**Table 1:** Summary of CMD and PaCS-MD

	CMD		oPaCS-MD		cPaCS-MD	
	CALB/W	CALB/I	CALB/W	CALB/I	CALB/W	CALB/I
Number of replicas	-	-	30	30	30	30
Number of trials	10	10	20	5	40	10
Average number of cycles per trial	-	-	49.6 ± 9.5	292.0 ± 64.2	22.5 ± 5.4	150.4 ± 46.1
Average simulation cost per trial (μs)	0.5	0.5	0.149 ± 0.028	0.876 ± 0.193	0.067 ± 0.016	0.451 ± 0.138
Total simulation cost (μs)	5.0	5.0	2.98	4.38	2.70	4.51

### Analysis by MSM

The analysis by MSM was conducted by PyEMMA2.5.12.^56^ For MSM, we employed all of the snapshots generated by the cPaCS-MD and oPaCS-MD trials. The inter-Cα distances between Arg309 and Leu144 (Dist1) and Ala146 and Val286 (Dist2) were chosen as the features to construct MSM (right bottom of Figure 2). Dist2 directly measures the lid opening, and Dist1 shows the effect of the lid opening on the β-hairpin situated outside α5. As the lid becomes more open, Dist1 and Dist2 tend to be shorter and longer, respectively. Using these two features, the snapshots sampled by PaCS-MD were clustered into 1000 microstates using k-means clustering^29^ with a k-means++ initialization strategy.^57^ We determined 1000 as the best number that can construct sufficiently fine-grained MSM with sufficient statistics based on the standard procedure of MSM.^29^ With this number, important conformational differences between microstates can be distinguished, such as multiple free-energy minima shown later with the obtained simulation data.

Each element of the transition probability matrix of MSM, ***T*** = {*T*_*ij*_ (τ)}, was calculated based on the features at time *t*(*x*(*t*)) and time *t* + τ(*x*(*t* + τ)) according to eq 1, where τ is the lag time and *S*_*i*_ and *S*_*j*_ represent the microstate before and after transition.^29^
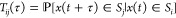
1The stationary probability of microstates, **π** = {*π*_*i*_}, is obtained by solving the eigenfunction in eq 2,^29^ and the free energy of each state was calculated using eq 3.^29^
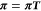
2
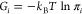
3The free-energy landscape was obtained by projecting the free energy into the two-dimensional space spanned by Dist1 and Dist2.

To obtain a more coarse-grained view of the open–close motion of the lid, macrostate analysis was conducted. The assignment of the macrostates was performed using PCCA++.^58^ The flux network between the obtained macrostates was analyzed using transition path theory (TPT).^29^

## Results

### Interactions between CALB and the Tricaprylin Surface

**Figure 3:**
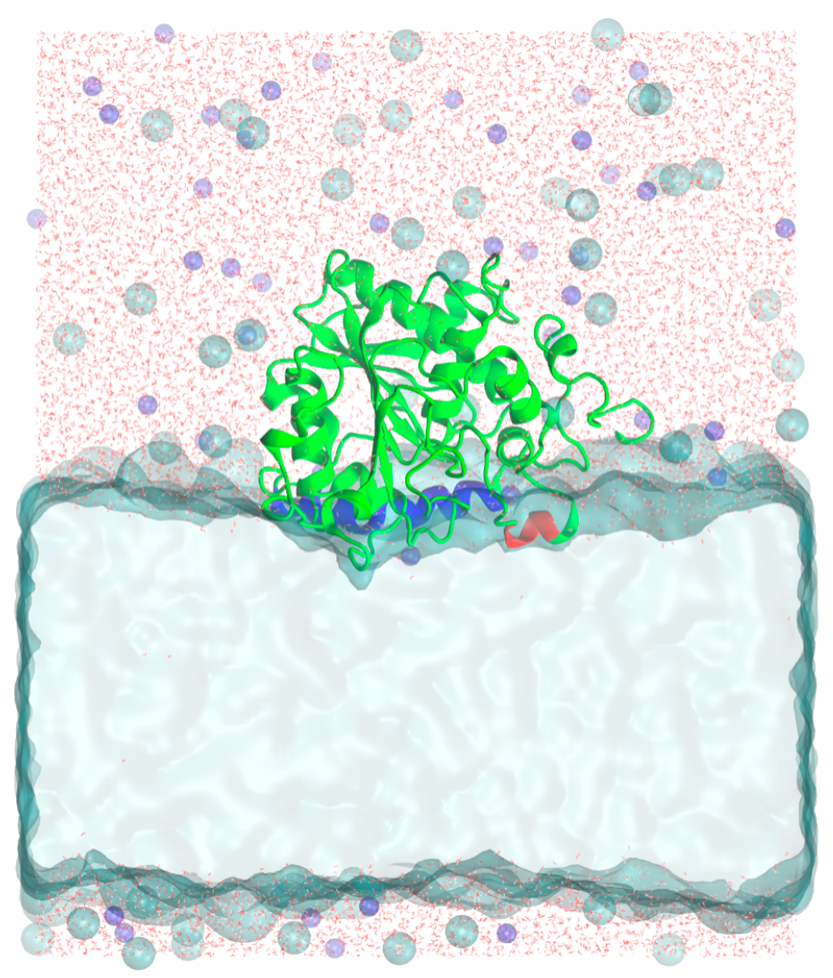
Representative snapshot from the CMD simulations showing CALB adsorbed onto the tricaprylin surface.

CMD simulations of the interface system (CALB/I) showed a strong interaction between CALB and the tricaprylin surface as follows. While CALB and the tricaprylin surface were initially separated by layers of water around 7 Å thickness with the lid region oriented to the surface (Figure 1C), CALB was adsorbed onto the tricaprylin surface at the end of all CMD simulations (Figure 3). This is expected because the lid region consists of mostly hydrophobic residues. Strong interaction between the lid and tricaprylin surface was also reported previously.^24,59^

### Conformational Dynamics of CALB Investigated by CMD and PaCS-MD

**Figure 4:**
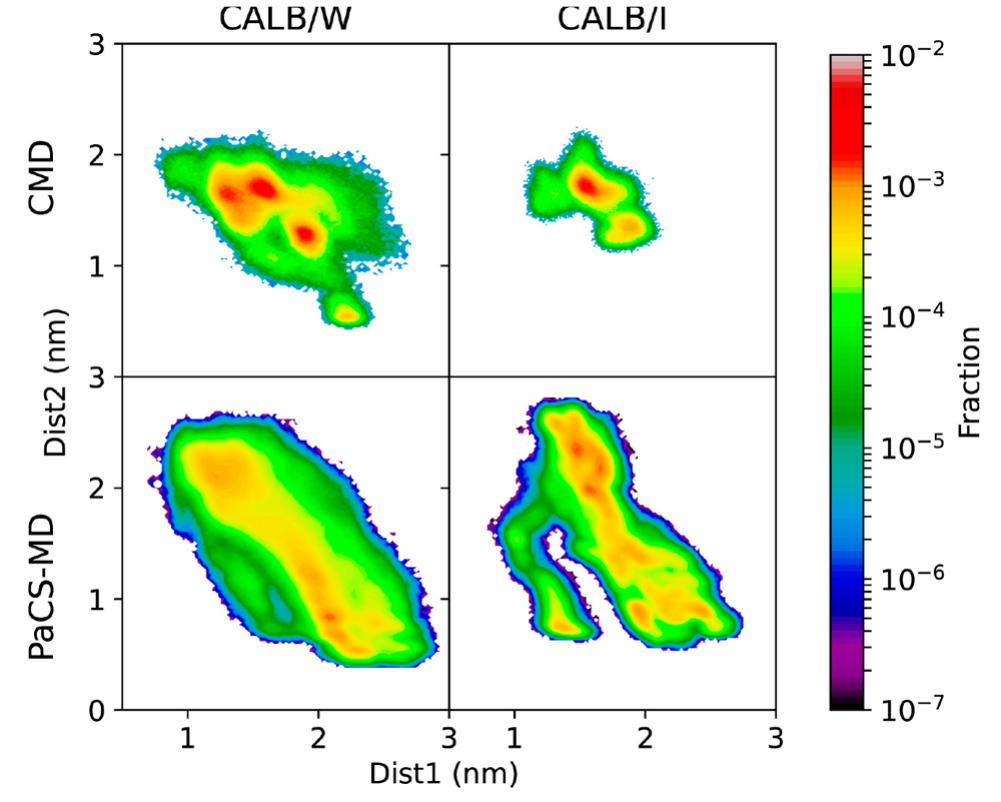
Projections of CMD and PaCS-MD trajectories (left and right) for both water and the interface system plotted in Dist1–Dist2 space.

To compare the conformational space sampled by CMD and PaCS-MD, we projected the obtained trajectories onto the Dist1–Dist2 space (Figure 4). In CALB/W, PaCS-MD sampled a much wider area than CMD and the difference is more apparent for CALB/I.

### MSM Analysis

MSMs for both systems were constructed by using Dist1 and Dist2 as the features, and the snapshots obtained by PaCS-MD were clustered into 1000 microstates using the k-means clustering approach (Methods). The appropriate lag time and validity of the MSMs were determined from the implied time scales (ITS) plot (Figure S1). Each MSM was validated by plotting the ITS over a series of lag times from 1 to 50 ps. The MSM is validated if the slowest time scale in the ITS plot reaches a certain constant value. The ITS plot showed that the slowest time scale reaches a relatively constant value after 30 ps for CALB/W and CALB/I, thus validating the MSM. For a typical long MD simulation, the smallest possible lag time is chosen to prevent any information lost due to a long lag time.^29^ However, a longer lag time assures better Markovianity than a shorter lag time.^29^ Since the PaCS-MD trajectories are short (100 ps each) in this work, the longest reasonable lag time is half of the MD length (50 ps). Therefore, to achieve better Markovianity for the MSM of PaCS-MD trajectories, the longest possible lag time should be chosen as long as the slowest implied time scale reaches a constant value. Therefore, a time of 50 ps was selected. The use of 100 ps MD and a 50 ps lag time was shown to reproduce experimentally determined free-energy values in many cases.^35−40^

**Figure 5:**
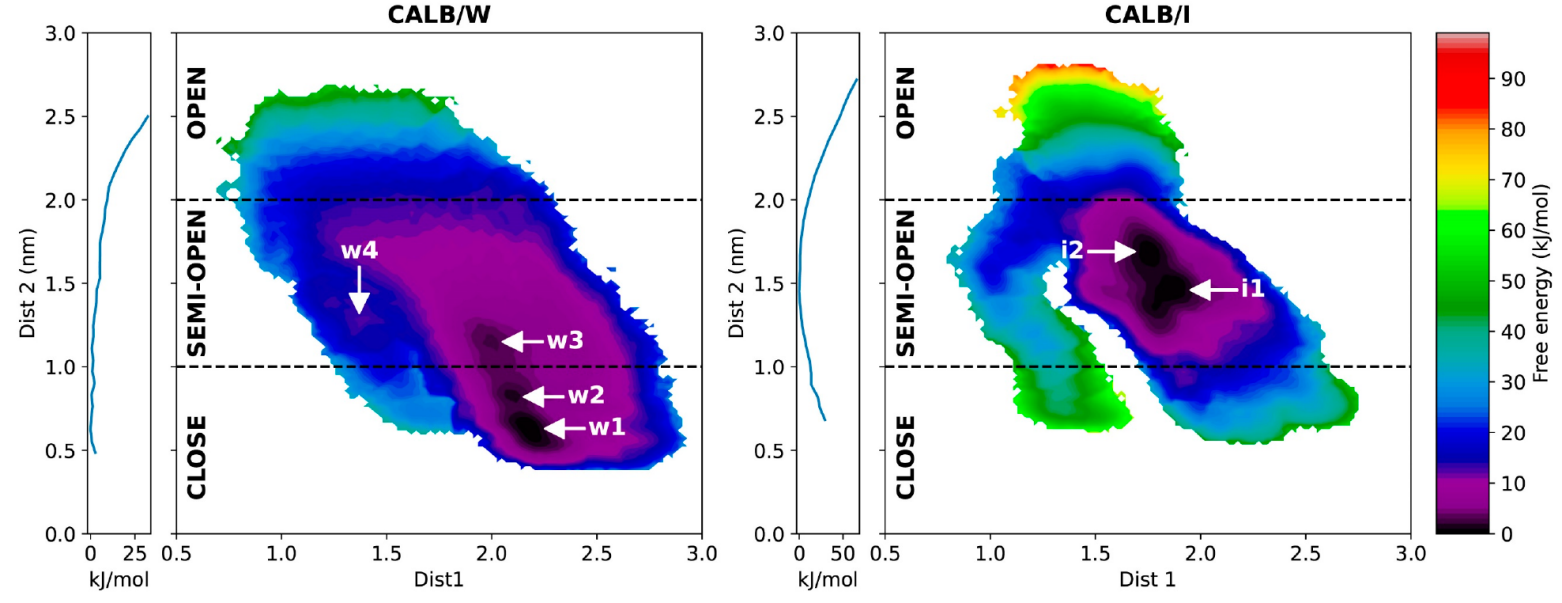
1D and 2D free-energy landscapes (FELs) of CALB/W and CALB/I obtained by PaCS-MD/MSM. The 1D FELs calculated along Dist 2 are shown as the vertical plots on the left side of the 2D FELs shown by color maps.

1D and 2D free-energy landscapes (FELs) of both systems (Figure 5) were calculated from the stationary distribution obtained from the transition probability matrix (eqs 2 and 3). Here, we categorized the CALB conformations into three types—closed: Dist2 ≤ 1.0 nm, semiopen: 1.0 nm < Dist2 < 2.0 nm, and open: Dist2 ≥ 2.0 nm. Molecular simulations tend to observe wider variations in interlid distances^18,21^ compared to those observed in crystals. (See Table S3, which will be explained later in the Discussion). To classify the open–close motion more in detail, we adopted a classification into three types of conformations similar to those in the literature^18,21^ rather than the classification only into two states, open and closed. The semiopen conformation in our definition is referred to as the open conformation in some crystallographic studies such as 1TCA and chains A of 5A71 and 5A6V in the PDB. The 1D FELs indicate that the global free-energy minimum exists in the closed state in CALB/W and that in CALB/I it is situated in the semiopen state. For CALB/W, the 2D FEL has four free-energy minima (*w1*–*w4*), numbered from the lowest free energy based on the 1D FEL. Three minima (*w1*–*w3*) are located close to each other, while the other (*w4*) is located far in the left area and has a much higher free energy. The global minimum *w1* and local minimum *w2* are classified into the closed conformation, while local minima *w3* and *w4* correspond to the semiopen conformation. On the other hand, the FEL of CALB/I shows only two minima located close to each other (*i1* and *i2*) which are also numbered from the lowest free energy based on 1D FEL. Both minima are categorized into the semiopen conformation. The depths of the two minima are comparable.

The FELs also indicate differences in low free-energy areas in the conformational space. The FEL of CALB/W covers a larger conformational space than CALB/I, which indicates more flexibility of the lid region in water. This is expected because CALB in the interface system is adsorbed on the tricaprylin surface, which restricts lid movement. The low free-energy areas around *i1* and *i2* are more restricted compared to the low free-energy areas around *w1*–*w3*, which indicates more stabilization or less flexibility of the lid region in CALB/I.

**Figure 6:**
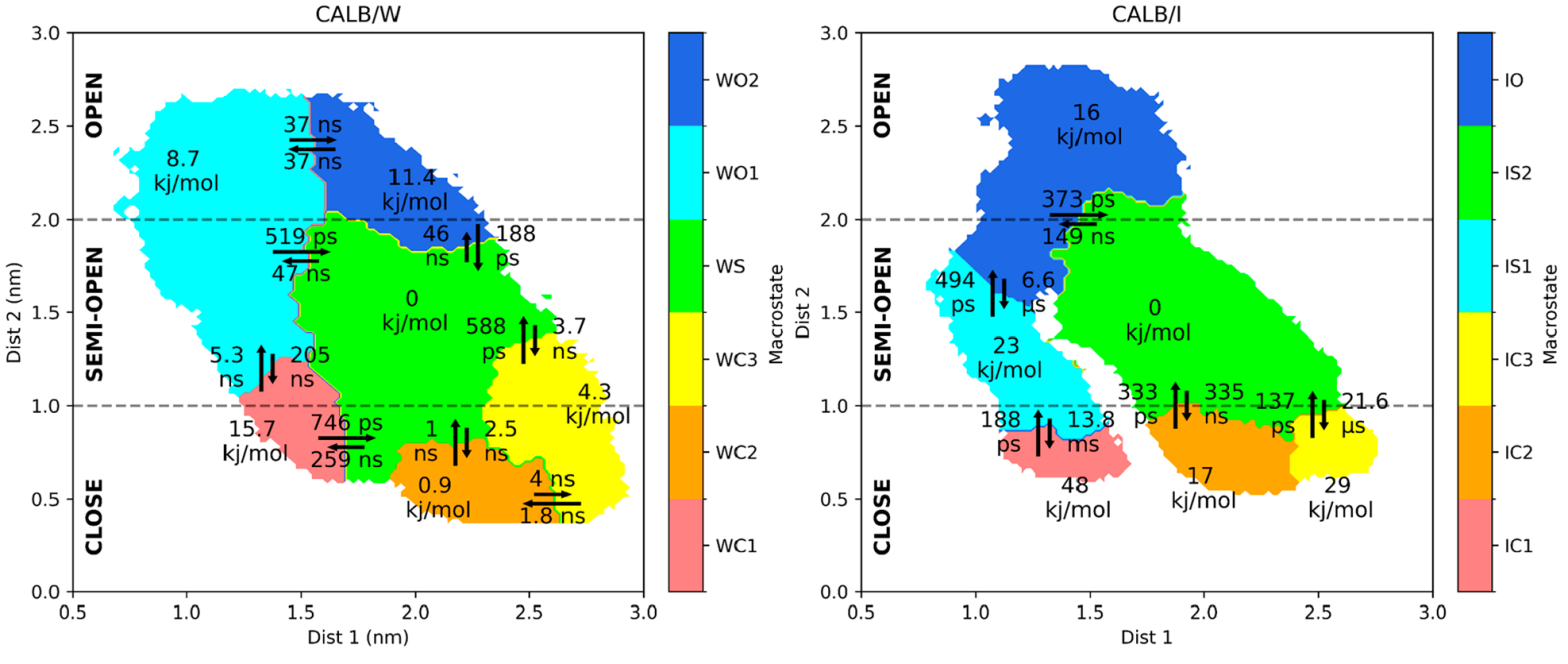
Macrostate assignment using PCCA++ for both CALB/W and CALB/I. PCCA++ results and MFPT between macrostates for CALB/W (left) and CALB/I (right). The transition time and free-energy difference are also indicated.

To obtain a more macroscopic view of FELs, macrostate assignment was performed by using PCCA++ (Figure 6). The number of macrostates for both systems was chosen so that the conformational space of the global minima is well-defined. This requirement was satisfied with six macrostates for both cases. In CALB/W, the conformational space was divided into three closed states (WC1, WC2, and WC3), one semiopen state (WS), and two open states (WO1 and WO2). The global free-energy minimum *w1* was assigned to WC2, while *w2* and *w3* were assigned to WS. The free energy of each macrostate was obtained as the sum of the probabilities of contained microstates converted to the free-energy value. Therefore, the free energy of the microstate containing the global free-energy minimum is not necessarily the lowest free-energy microstate as the macrostates can also include high free-energy regions. Actually, this is the case for WC2 that contains *w1*, but its free-energy value is slightly higher than that of WS. Transition times to WS from the other macrostates tend to be faster (≤1 ns) than transitions from WS to the other states (>2.5 ns), but the transitions between WC2 and WS are comparable. Transition times from the semiopen state (WS) to the open states (WO1 and WO) are 47 and 46 ns, respectively. From WS, the transitions to WC2 and WC3 are 2.5 and 3.7 ns, respectively, which shows that the transition from the semiopen to the closed state is one order of magnitude faster than that to the open state.

For CALB/I, the conformational space was divided into three closed states (IC1, IC2, and IC3), two semiopen states (IS1 and IS2), and one open state (IO). Both *i1* and *i2* were assigned to IS2, which shows the lowest free energy, and the other macrostates are situated in higher-energy areas of the FEL. In this case, the lowest free-energy minimum *i1* is included in IS2, whose free energy is the lowest among the macrostates. The transition time from the semiopen state (IS2) to the open state (IO) is slower (149 ns) than those in water. This is probably due to the strong interaction between the lid and tricaprylin.

### Analysis by Transition Path Theory

**Figure 7:**
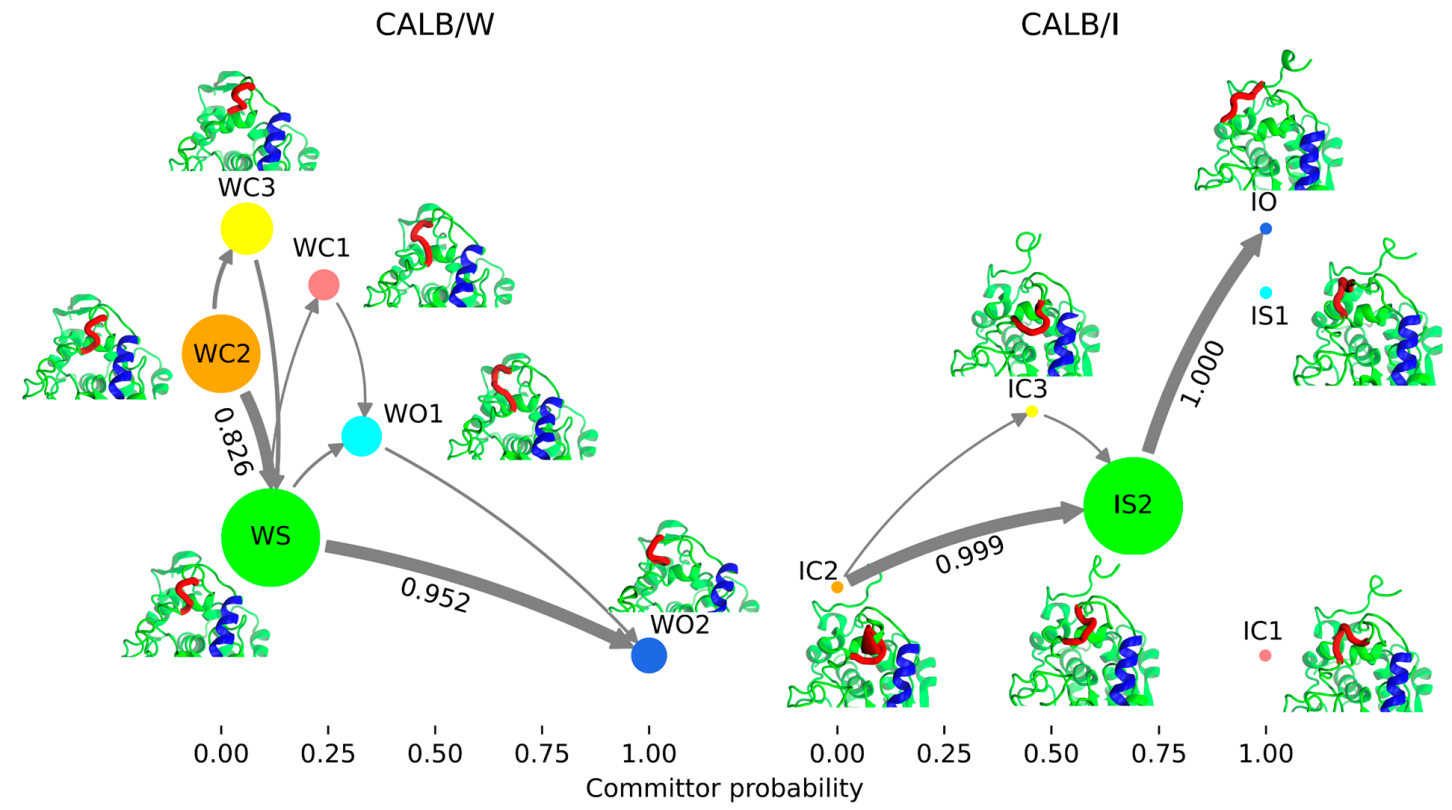
Flux network of from the closed to open conformation for CALB/W and CALB/I. The macrostates were arranged by their committor probabilities along the abscissa. The values near the arrows are the flux fractions of each transition between macrostates. For CALB/W, the circle area is proportional to −1/(ln π) of the corresponding macrostate. For CALB/I, the ratio of the circled area between IS2 and other macrostates is fixed at 100:1 for clarity due to the stationary probability of IS2 which is over 0.99.

To investigate the main transition pathway from the closed to the open state, the flux network was calculated by the coarse-grained MSM obtained by PCCA++ and TPT (Figure 7). For CALB/W, WC2 and WO2 were selected as the starting and end points, respectively, and IC2 and IO were selected as the starting and end points for CALB/I, respectively.

The flux network of CALB/W showed several pathways from the closed (WC2) to open (WO2) state, and the major pathway is WC2 → WS → WO2. In CALB/I, the closed (IC2) to open (IO) state pathways are more restricted, the major pathway being IC2 → IS2 → IO. Similar to CALB/I, the closed to open transition goes through the semiopen macrostate, IS2. IC1 and IS1 located in the left arm of the free-energy landscape (Figure 6) are disconnected from the network, indicating that the closed to open transition does not go through these macrostates. Interestingly, the semiopen macrostate of each system has a different committor probability. The committor probability of WS in CALB/W was 0.12, while that of IS2 in CALB/I was 0.7. In this particular case, a larger committor probability means that the probability of transition to the open state is higher than that of transition to the closed state and vice versa. Therefore, CALB in the semiopen state tends to make a transition to the closed state in water but moves to the open state in the interface. This result is consistent with the aforementioned transition time results.

### Interaction Probabilities along the Major Pathways

To identify important residues that are responsible for CALB conformational dynamics in both systems, interaction changes in the lid region were quantified along the major pathways, WC2 → WS → WO2 for CALB/W and IC2 → IS2 → IO for CALB/I. The interactions were analyzed by calculating the residue–residue contacts and hydrogen bonds among residues 135–151 and 261–293 including the α5 and α10 helices and several additional residues along both ends.

Contacts and hydrogen bonds between residue pairs were identified based on simple geometric criteria. In the analysis of residue–residue contacts, residue pairs were considered to be in contact if any of their heavy atoms are separated by less than 5.0 Å. The residue–residue hydrogen bonds were identified among an acceptor heavy atom (A), a donor heavy atom (D), and a donor hydrogen atom (H) if the distance A–D is less than 3.0 Å and the angle A–H–D is larger than 135°. To calculate the average numbers of contacts and hydrogen bonds per macrostate, sample structures were obtained by extracting 25 structures from each microstate in the macrostate. Then, the average numbers of contacts and hydrogen bonds were calculated, weighted by probabilities of microstates π and shown in Table 2.

**Table 2:** Average Numbers of Contacts and Hydrogen Bonds in the Lid Region along the Major Pathways, WC2 → WS → WO2 for CALB/W and IC2 → IS2 → IO for CALB/I

	Contacts			Hydrogen bonds		
CALB/W	WC2	WS	WO2	WC2	WS	WO2
	171.2	164.6	160.4	9.7	9.7	9.3
CALB/I	IC2	IS2	IO	IC2	IS2	IO
	171.4	169.1	164.6	10.4	12.0	12.0

For both systems, the numbers of contacts in the closed states are almost the same, but more contacts were lost upon the transitions to the semiopen and open states in CALB/W. Unlike the number of contacts, the number of hydrogen bonds in the CALB/I closed states is slightly more than that in CALB/W. This is probably due to the compensation of the lost hydrogen bonds between CALB and water in the interface by intramolecular hydrogen bonds in the lid region. Additionally, the number of hydrogen bonds increased upon the transition from the closed to semiopen state in CALB/I but did not change upon the transition from the semiopen to open state. More hydrogen bonds in IS2 than in IC2 may contribute to the higher stability of the semiopen state compared to that of the closed state in the interface system. In the case of CALB/W, the number of hydrogen bonds did not change in the transition from the closed to semiopen state and slightly decreased upon the semiopen to open transition. The loss of intramolecular hydrogen bonds upon the second transition might be compensated for by the formation of CALB–water hydrogen bonds.

To analyze the interaction changes on a residue–residue basis, the contact probability *p*_*k*_^*ij*^ for each identified residue pair *i* and *j* and each microstate *k* was calculated from 25 structures. For each macrostate, the contact probability (*P*^*ij*^) was calculated using the following formula:
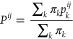
4For the hydrogen bond analysis, the hydrogen bond probability was calculated using a procedure similar to that for the contact probability. To quantify the changes in contact and hydrogen bond probabilities caused by the transition from the initial macrostate *B* and final macrostate *C*, Δ*P*^*ij*^ = *P*^*ij*^(*C*) – *P*^*ij*^(*B*) was calculated. In this case, positive Δ*P*^*ij*^ corresponds to an increase in contact and hydrogen bond probabilities after the transition while negative Δ*P*^*ij*^ indicates a decrease in contact and hydrogen bond probabilities after the transition. In this analysis, only contacts and hydrogen bonds with |Δ*P*^*ij*^| > 0.2 are considered.

The number of identified residue–residue contacts and hydrogen bonds with significant probability changes along the main transition pathways (WC2 → WS → WO2 for CALB/W and IC2 → IS2 → IO for CALB/I) is shown in Table S1. Different change patterns can be observed between CALB/W and CALB/I in the closed to semiopen transitions. In the IC2 → IS2 transition of CALB/I, 13 contact losses were mostly compensated for by 10 contact gains, which indicates significant rearrangements of residue–residue contacts in the transition. In contrast, the contacts were simply lost in the WC2 → WS transition of CALB/W. In the semiopen to open transitions in both water and interface systems, the contact loss was partially compensated for by the contact gain.

As already shown in Table 2, the gain of hydrogen bonds mainly occurred upon the IC2 → IS2 transition in CALB/I, which is also seen in the analysis of Δ*P*. To take a closer look at the interaction changes upon this transition, Table S2 lists the residue pairs with |Δ*P*| > 0.2 for contacts and hydrogen bonds. The result clearly shows an interaction gain between the α5 and α10 regions upon this transition. The contacts increased between Ala148 near α5 and Asn292/Gln291 near α10, and one hydrogen bond was formed between the main-chain oxygen of Ala148 and the side-chain HN of Asn292 (Figure S2). This transition accompanied the loss of hydrogen bonds between the side chains of Asp145 and Ser150 within around α5. These interaction changes stabilize the semiopen conformation in CALB/I.

## Discussion

The results of this research indicate that the tricaprylin surface affects the conformational dynamics of the CALB by shifting the global free energy minimum from the closed conformation in water to the semiopen conformation in the interface system. These results are consistent with experimental evidence for CALB interfacial activation when CALB is immobilized on highly hydrophobic silanized beads.^18^ The shift of the global free energy minimum is accompanied by deeper minima in CALB/I than those in CALB/W, which implies less flexibility in the interface system. This is consistent with the low flexibility of CALB in organic solvent.^60^ It should be noted that the stabilization of the semiopen state in CALB/I is accompanied by an increase in the free energy of the open state compared to that in CALB/W as shown in Figure 5, which indicates that the transition from the semiopen to open state in CALB/I is slower than that in CALB/W.

However, it is still unclear whether substrates first bind to CALB in the semiopen conformation or if the open conformation is required for the initial binding. If the former is true, then the tricaprylin interface can facilitate the initial binding, which may also contribute to the interfacial activation. In the latter case, the free-energy difference between the open and semiopen states in the interface system is expected to be lowered by certain mechanisms to achieve the interfacial activation. To consider the initial binding of substrates, we examined the available complex structures of CALB in the Protein Data Bank. First, we examined the crystal structure of CALB with Tween 80 (PDB id: 1LBT)^60^ having only one ester bond which can react with the CALB catalytic triad. In this structure, Tween 80 is fully bound to CALB in close proximity to the catalytic triad, which is an appropriate position for catalysis to occur. Also, CALB takes a semiopen conformation according to our definition (Dist2 = 15.5 Å), respectively. Therefore, the semiopen conformation is suggested to be sufficient for the binding of Tween 80. We also considered the crystal structure of CALB complexed with tributyrin but inhibited by the active-site modification with ethyl phosphonate (PDB id: 6TP8).^61^ Similar to tricaprylin, tributyrin is a triglyceride containing three ester bonds that can react with a CALB catalytic triad. In this structure classified as a semiopen conformation (Dist2 = 15.4 Å), tributyrin is located in the limbus region of the CALB catalytic site, whose distance from catalytic Ser105 is 10 Å. This result implies that the initial binding of tributyrin to CALB can occur in the semiopen state, but further relocation of tributyrin to the “full binding” state might be needed because the 10 Å distance may be too far for catalysis. Triglyceride has three ester bonds, which might require a larger space around the CALB catalytic triad. If the full binding should accompany a conformational transition from the semiopen to open states, then it is expected that lowering the free-energy difference between the semiopen and open states is induced by the initial binding of tributyrin for the interfacial activation. In relation to our results, we speculate that the stabilization of the semiopen state in CALB/I could facilitate faster initial binding of tricaprylin to CALB. If a transition from the initial binding to the full binding is required for tricaprylin, then relatively high free energy of the open state in CALB/I is expected to be lowered by the interactions between CALB and tricaprylin generated by the initial binding. To investigate this hypothesis, we plan to conduct new simulations as the next project.

Stauch et al. pointed out a possible role of the salt bridge between Asp145 and Lys290 in alternating the conformation.^19^ They determined the crystal structures of CALB with (PDB id: 5A6V) and without xenon (5A71) and found that a salt bridge between Asp145 and Lys290 is formed in the closed conformations (5A6V: chain B and 5A71: chain B). “Chain” is omitted hereafter, while it is not formed in the open conformation (5A6V: A and 5A71: A). The values of Dist2 are 15.9 Å (5A6V: A), 9.8 Å (5A6V: B), 16.0 Å (5A71: A), and 10.1 Å (5A71: B), and thus 5A6V: B is categorized into the closed state and the others are classified into the semiopen state in our definition. We examined 28 distinct CALB conformers in 22 deposited structures in the PDB (Table S3), and the Asp145–Lys290 salt bridge is formed only in 5A71: B (Dist2 = 10.1 Å) and 5A6V: B (9.8 Å). Dist2 is relatively close to 5A6V: B and 5A71: B in a few cases (4K6H: B 11.4 Å, 6J1P 11.3 Å, 6J1Q 11.4 Å, and 6J1S 10.1 Å), but the salt bridge is not observed. All of the CALBs in these cases include mutations, which might also affect the stability of “near” closed conformations.

We also calculated the probability of Asp145–Lys290 salt bridge formation during the simulation (Table S4). It should be noted that both Asp145 and Lys290 are treated as charged in our simulations. This salt bridge was not formed in WC1, WC2, and WC3 and formed only with a very small probability (0.1%) in WS in CALB/W. The probability of Asp145–Lys290 salt bridge formation was 4.4% in IC3 of CALB/I, which is much higher than that in CALB/W. Since IC3 is categorized as the closed state, the formation of Asp145–Lys290 could contribute to the stabilization of the closed state as previously reported.^19^ To examine if this tendency is caused by the specific force field, we conducted ten independent MD simulations with the CHARMM36m force field in TIP3P water.^62^ After equilibration, 200 ns MDs were conducted, and the last 100 ns trajectories were used to calculate the probability of the salt bridge formation, which was significantly higher (19%). This shows some force-field dependence of the salt bridge formation. However, the simulations with both AMBER ff19SB/OPC and CHARMM36m/TIP3P indicate a relatively low probability of the salt bridge formation in water. This implies that the results might also be affected by environmental differences between the solution and crystal. The salt bridge formation is expected to stabilize the closed state more, as shown in 5A6V: B, but our results suggest that even without the salt bridge formation the closed state is still stable in water.

## Conclusions

In this article, PaCS-MD was employed to sample the conformational dynamics of CALB both in water and at the water–tricaprylin interface. The analysis of PaCS-MD trajectories using MSM indicated that the closed state is the most stable in the water system but the most stable conformation in the interface system shifts to the semiopen state. The conformational shift could facilitate faster initial binding of the substrate. These effects could explain the energetics and kinetics origin of the previously reported interfacial activation of CALB. These findings could help expand CALB applications toward a wide variety of substrates.

## Supporting Information Available

Numbers of residue–residue contacts and hydrogen bonds with significant probability changes, residue pairs for contacts and hydrogen bonds upon the IC2 → IS2 transition in CALB/I, list of CALB structures in the Protein Data Bank, probabilities of salt bridge formation between Asp145 and Lys290, implied time scale plots for CALB/W and CALB/I, and visualization of the key residues (PDF)

## Abbreviations

CALB: *Candida antarctica* lipase B
MD: molecular dynamics
PaCS-MD: parallel cascade selection molecular dynamics
MSM: Markov state model
PDB: protein data bank
CALB/W: CALB in water
CALB/I: CALB on the interface
