# Supplementary material for: Energetic and Kinetic Origins of CALB Interfacial Activation Revealed by PaCS-MD/MSM
Source: J Phys Chem B. 2023 Aug 10;127(34):7431–41. doi: 10.1021/acs.jpcb.3c02041 (PMC10476181; doi:10.1021/acs.jpcb.3c02041)

Supporting Information for

Energetic and Kinetic Origin of CALB Interfacial Activation Revealed by PaCS-MD/MSM

*Tegar N. Wijaya[1,2] and *Akio Kitao[1]*

[1] School of Life Science and Technology, Tokyo Institute of Technology. 2-12-1 Ookayama, Meguro-ku, Tokyo 152-8550, Japan. Tel +81-3-5734-3373

[2] Department of Chemistry, Universitas Pertamina, Jl. Teuku Nyak Arief, Simprug, Jakarta 12220, Indonesia. Tel +62 812-1149-9919

*Corresponding author: Akio Kitao, School of Life Science and Technology, Tokyo Institute of Technology, M6-13, 2-12-1 Ookayama, Meguro-ku, Tokyo 152-8550, Japan

E-mail: akitao@bio.titech.ac.jp

Table S1. The numbers of residue-residue contacts and hydrogen bonds with significant probability changes upon the closed to semi-open and semi-open to open transitions in CALB/W and CALB/I. Contacts and hydrogen bonds shown in bold fonts are listed in Table S2.

	$\Delta P < -0.2$	$0.2 \leq \Delta P$
Contacts		
CALB/W, WC2 → WS	14	0
CALB/W, WS → WO2	11	4
CALB/I, IC2 → IS2	**13**	**10**
CALB/I, IS2 → IO	14	6
Hydrogen bonds		
CALB/W, WC2 → WS	1	2
CALB/W, WS → WO2	2	1
CALB/I, IC2 → IS2	**1**	**3**
CALB/I, IS2 → IO	0	2

Table S2. Residue pairs with $|\Delta P| > 0.2$ for contacts and hydrogen bonds upon the IC2 → IS2 transition in CALB/I. The interactions shown in bold fonts indicate those especially contribute to the transition.

Contact residue pairs			
$\Delta P > 0.2$		$\Delta P < -0.2$	
Residue pairs	ΔP	Residue pairs	ΔP
**Ala148-Asn292**	**0.84**	Leu144-Ile285	-0.88
**Ala148-Gln291**	**0.76**	Pro143-Val286	-0.84
Pro262-Ala275	0.59	Leu144-Ala151	-0.83
Val149-Asn292	0.52	Pro143-Ala282	-0.81
Asp145-Ser150	0.45	Pro143-Ile285	-0.79
Leu147-Asn292	0.38	Leu147-Val286	-0.69
Val149-Gln291	0.37	Leu144-Pro289	-0.68
Leu266-Ala274	0.32	Leu147-Lys290	-0.67
Ala151-Ile285	0.32	Leu144-Val286	-0.60
Ala283-Gly288	0.28	Leu144-Lys290	-0.55
		Asp145-Val149	-0.41
		Leu144-Ser150	-0.40
		Ile285-Pro289	-0.34
Hydrogen bond residue pairs			
$\Delta P > 0.2$		$\Delta P < -0.2$	
Residue pairs	ΔP	Residue pairs	ΔP
**Ala148-Asn292**	**0.42**	**Asp145-Ser150**	-0.43
Ala283-Ala287	0.25		
Gly142-Ala146	0.21		

Table S3 List of CALB structures in Protein Data Bank, Dist2, classification of states, and salt bridge formation.

PDB id	Chain	Sequence	Dist2 (Å)	State	Salt bridge
1TCA		Wild type	15.6	Semi-open	No
1TCB		Wild type	15.8	Semi-open	No
1TCC		Wild type	15.7	Semi-open	No
1LBT		Wild type	15.5	Semi-open	No
1LBS		Wild type	16.0	Semi-open	No
3W9B	A	Wild type	15.4	Semi-open	No
3W9B	B	Wild type	15.3	Semi-open	No
4K6H	A	1 mutation	19.1	Semi-open	No
4K6H	B	1 mutation	11.4	Semi-open	No
4K5Q		2 mutations	19.3	Semi-open	No
4K6K	A	1 mutation	Helix 5 missing		-
4K6K	B	1 mutation	Helix 5 missing		-
4K6G		Wild type	Helix 5 missing		-
4ZV7		Wild type	15.5	Semi-open	No
5A71	A	Wild type	16.0	Semi-open	No
5A71	B	Wild type	10.1	Semi-open	Yes
5A6V	A	Wild type	15.9	Semi-open	No
5A6V	B	Wild type	9.8	Closed	Yes
6TP8	A	Wild type	15.3	Semi-open	No
6TP8	B	Wild type	15.4	Semi-open	No
6J1T		4 mutations	18.9	Semi-open	No
6J1P		4 mutations	11.3	Semi-open	No
6ISQ		7 mutations	19.8	Semi-open	No
6ISR		7 mutations	19.5	Semi-open	No
6ISP		6 mutations	12.2	Semi-open	No
6J1Q		4 mutations	11.4	Semi-open	No
6J1R		4 mutations	19.4	Semi-open	No
6J1S		6 mutations	10.1	Semi-open	No

Table S4. Probabilities of salt bridge formation between Asp145 and Lys290 in each macrostate.

CALB/W		CALB/I	
Macrostate	Probability (%)	Macrostate	Probability (%)
WC1	0.0	IC1	0.0
WC2	0.0	IC2	0.0
WC3	0.0	IC3	4.4
WS	0.1	IS1	0.0
WO1	0.0	IS2	0.1
WO2	0.0	IO	0.0

Figure S1. Implied timescale plots for CALB/W and /I.

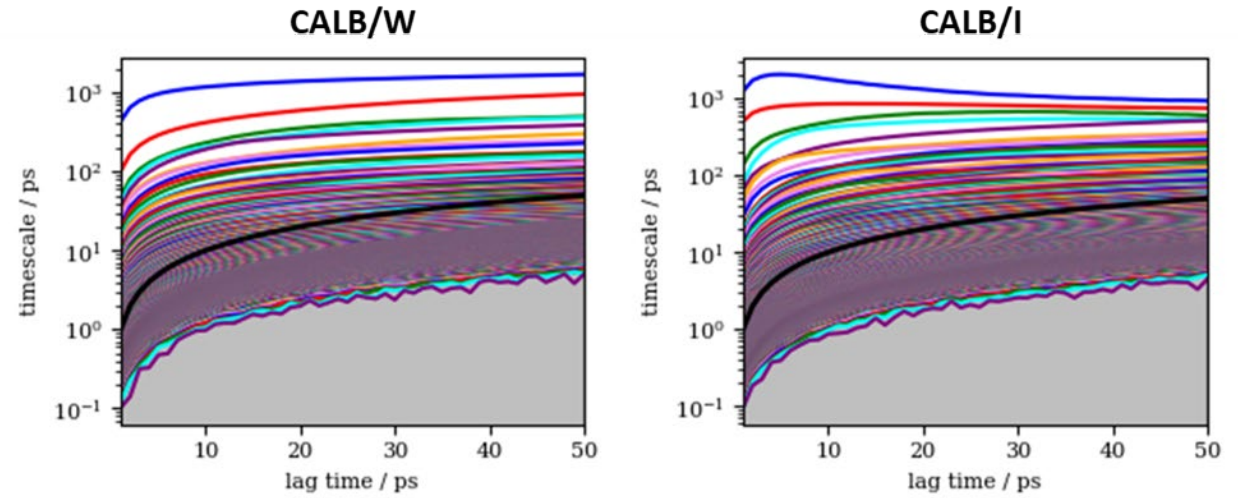

Figure S2. Visualization of the key residues identified in Table S2.

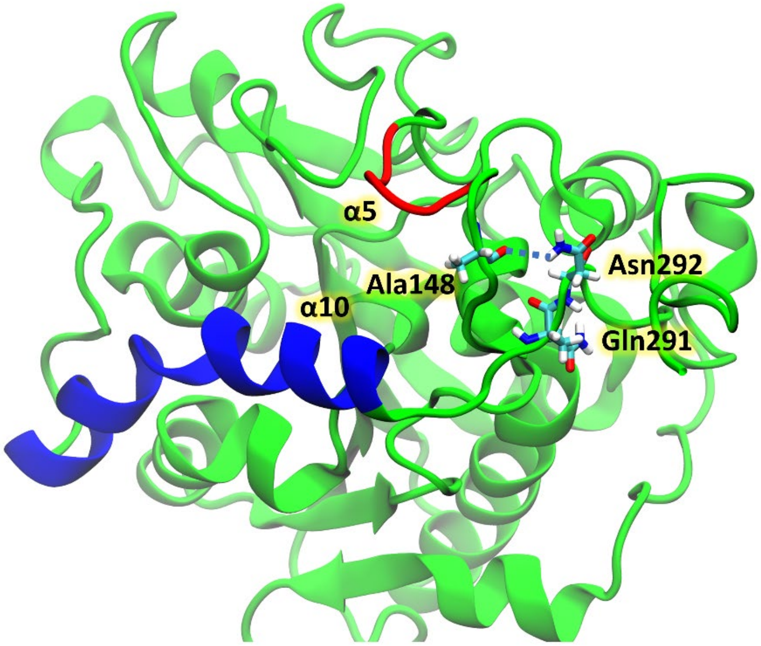

Supplement: Supplementary file 1 — jp3c02041_si_001.pdf [file jp3c02041_si_001.pdf]
